# Analyses of inter-rater reliability between professionals, medical students and trained school children as assessors of basic life support skills

**DOI:** 10.1186/s12909-016-0788-9

**Published:** 2016-10-07

**Authors:** Stefanie Beck, Bjarne Ruhnke, Malte Issleib, Anne Daubmann, Sigrid Harendza, Christian Zöllner

**Affiliations:** 1 Department of Anaesthesiology, University Hospital Hamburg-Eppendorf, Martinistr. 52, 20246 Hamburg, Germany; 2 The Medical Faculty of the University Hamburg, Martinistr. 52, 20246 Hamburg, Germany; 3 Department of Medical Biometry and Epidemiology, University Medical Center Hamburg-Eppendorf, Martinistr. 52, 20246 Hamburg, Germany; 4 Department of Internal Medicine, University Medical Center Hamburg-Eppendorf, Martinistr. 52, 20246 Hamburg, Germany

**Keywords:** Basic life support, Resuscitation, Assessment, Inter-rater reliability, Peer assessors

## Abstract

**Background:**

Training of lay-rescuers is essential to improve survival-rates after cardiac arrest. Multiple campaigns emphasise the importance of basic life support (BLS) training for school children. Trainings require a valid assessment to give feedback to school children and to compare the outcomes of different training formats. Considering these requirements, we developed an assessment of BLS skills using MiniAnne and tested the inter-rater reliability between professionals, medical students and trained school children as assessors.

**Methods:**

Fifteen professional assessors, 10 medical students and 111-trained school children (peers) assessed 1087 school children at the end of a CPR-training event using the new assessment format. Analyses of inter-rater reliability (intraclass correlation coefficient; ICC) were performed.

**Results:**

Overall inter-rater reliability of the summative assessment was high (ICC = 0.84, 95 %-CI: 0.84 to 0.86, *n* = 889). The number of comparisons between peer-peer assessors (*n* = 303), peer-professional assessors (*n* = 339), and peer-student assessors (*n* = 191) was adequate to demonstrate high inter-rater reliability between peer- and professional-assessors (ICC: 0.76), peer- and student-assessors (ICC: 0.88) and peer- and other peer-assessors (ICC: 0.91). Systematic variation in rating of specific items was observed for three items between professional- and peer-assessors.

**Conclusion:**

Using this assessment and integrating peers and medical students as assessors gives the opportunity to assess hands-on skills of school children with high reliability.

## Background

According to the International Liaison Committee on Resuscitation (ILCOR)-guidelines of 2010 the most important determinant of survival from sudden cardiac arrest is the presence of a trained lay rescuer who is ready, willing, and able to act [1].

All over the world initiatives aim to increase the ability and awareness of potential lay rescuers. Initiatives focus on providing adequate training material and recommend to introduce basic life support (BLS) training in schools [2]. Different training concepts e.g. standard instructor-led hands-on training in groups and self-instruction with training videos have been shown to improve BLS skills of school children [3]. It is difficult to compare the effectiveness of the different training concepts because there is no uniform assessment [4]. The ILCOR-guidelines 2010 outline the need for further research on optimizing assessment of CPR skills to support individual learning by providing feedback (formative assessment). It also provides the opportunity to compare the effectiveness of different training formats (summative assessment) [3]. However, assessing BLS skills in a school setting is challenging because assessment in this context takes up a lot of manpower. Because mannequins with included digital skill-reporters are expensive and their transport complex, cost-effective and portable MiniAnne mannequins (Laerdal®) are widely used for BLS hands-on training of school children.

We designed a study to improve the feasibility to assess BLS skills at schools. By including medical students and trained school children (peers) as assessors we reduced the personnel requirement of professional assessors.

The two aims of the study were to develop a context-appropriate assessment of hands-on BLS skills of school children at schools and to test the inter-rater reliability when using professionals, medical students and trained school children as assessors.

## Methods

### Setting

This validation study was associated with a study investigating the effectiveness of peer-led hands-on BLS training for school children compared with professional-led training [5]. The study took place at eight schools in Hamburg, Germany, during the awareness-weeks of the national campaign “einlebenretten” (“save one life”) in September 2013 and 2014 and the local campaign “Hamburg rettet Leben” (“Hamburg saves lifes”) in March 2014. The assessment was part of an educational event at the schools and included school children of grades 7 to 10 (twelve to sixteen years old). The educational event lasted three hours and consisted of three parts. Part one was a 30-min lecture based on a lecture for the school children (available at the platform “www.einlebenretten.de”) provided by a physician. Part two consisted of 45 min hands-on training in basic life support (BLS) in groups of 10–14 school children with two instructors using “MiniAnne” (Laerdal®). Every group used 4 MiniAnne mannequins and every child practiced all skills evaluated with the formative-assessment in two-rescuer-scenarios. The third part was the “Assessment”.

### Assessment

For the practical assessment, we used a standardised three minutes/two-rescuer scenario assessed by a checklist. The examinees were supposed to perform the initial check for responsiveness and breathing and start CPR. They were supposed to tell an assistant rescuer to call the emergency medical service (EMS) and get an automated external defibrillator (AED) and attach it to the mannequin. The assistant rescuer was a child assessed immediately before. We set up numbered assessment-sites (up to eight), each with a MiniAnne and two examiners. The assessors were mixed based on alphabetical lists. We classified professionals and medical students as reference-assessors and tried to combine one reference-assessor with one peer-assessor. At some schools there were more assessment-sites than professional assessors. In this case we used assessment pairs consisting of two peers. If there were more professional assessors we combined one professional and one student or two professional assessors. The school children were randomly assigned to the assessment-sites, read the case vignette, and started the assessment upon an acoustic signal.

The assessors judged independently based on a structured assessment checklist consisting of 15 items. All school children received feedback about their performance based on 15 items (formative assessment) from a professional-instructor after their assessment. Eight items of this assessment were relevant to either “pass” or “fail” the assessment. These eight items (summative assessment) were used to compare training outcomes between the peer-led and professional-led training.

### Assessors

Trained school children (peers), professional instructors and medical students acted as instructors and assessors at the school event. Peers were school children of the participating schools who were trained beforehand. The training of the peers included two three-hour training sessions provided by two anaesthesiologists who were experienced in instructing medical students in BLS. The first session focused on a knowledge-based access to cardiac arrest and resuscitation as well as hands-on training in BLS. During the second session the peers were taught the 4-step-approach (sequence of teaching steps recommended in the ERC instructor manual) [6] and practiced to rate BLS-performance based on our formative assessment-checklist.

The potential peers were recruited by their teachers. Participation was offered to all school children of a class/course by their teachers. The school children participated voluntarily.

The medical students were in their final year of study which consists of four months in internal medicine, four months in surgery and four months in a subject of their choice. All students in our study had chosen their elective in the clinic of anaesthesiology of the University Hospital Hamburg-Eppendorf and participated voluntarily in the educational events for the school children.

The professional instructors were recruited from the staff of the Clinic of Anaesthesiology at the University Hospital Hamburg-Eppendorf. All of the professionals were experienced in training others in BLS and also volunteered to participate in the training at the schools. All assessors were trained up to 2 weeks in advance to use the formative assessment-checklist. The training was a group-training for 5–20 assessors. BLS skills were rated during three CPR-scenarios by all assessors and a reference rater (ERC-ALS-instructor). The individual results were discussed and aligned with the results of the reference-rater (frame-of-reference training) [7].

### Assessment-instrument

#### First step

We developed a formative assessment-checklist (Table 1) based on the learning objectives for the educational events and providing a structured guide for giving feedback during the hands-on training and after the practical examination. The instructions “check-call-compress” of the initiative “einlebenretten” formed the basis of the formative assessment to maintain structure during the educational event. In a stepwise process, formative assessment was developed by listing all instructions of “einlebenretten” [8] and the ERC-BLS-algorithm [9] and uniting the items. We added parameters, which were based on international consensus to define expected dimension of performance. To rate sufficient compression depth we used the “klicking” of the MiniAnne mannequin indicating compression depth between 42.5–57.5 mm. The compression rate was counted using a tapping beat-counter (free application for android: *BPM Tap* by Ignace Maes and free application for i-phone: *BPM*_*counter* by Torsten Klaus). Hand-activated stop-watch measurements were used to measure the lengths of time until starting chest-compression and the lengths of time of interruptions of chest-compression.

**Table 1:** Steps to develop formative assessment

Instructions for BLS according to “einlebenretten”	ERC-instructions for effective BLS including AED	Formative assessment	Specified dimension of performance
Ask the victim loudly: “can you hear me?”	Unresponsive?	Speaks to the victim and shakes his shoulders	
Shake his shoulders: no reaction?	Call for help	Calls for help	
Pay attention to breathing: No breathing or no normal breathing	Open airway: Not breathing normally?	Checks if the victim is breathing normally	Opens airway and comes close
Call 112 or send to call 112	Call 112	Calls or sends to call 112	
Compress hard and fast	30 chest compressions		
Start immediately	Start chest compression	Starts chest-compression as soon as possible	Within the first 30 s
Place the heel of one hand in the center of the chest, place the heel of the other hand on top	Place the heel of one hand in the center of the chest, place the heel of the other hand on top	Has the right compression point	Center of the chest
Interlock your fingers, keep your arms straight, position vertically above the compression point	Interlock your fingers, keep your arms straight, position vertically above the victims chest	Has the right position	
Press the sternum 5–6 cm down	Press down on the sternum at least 5 cm (but not exceeding 6 cm)	Has the right compression depth	5-6 cm depth, Mini-Anne clicking min. 4 of 5 compr.
	Allow full chest recoil	Releases all the pressure from the chest	
Compress 100–120 times per minute	Repeat at a rate of at least 100 min¯^1^ (but not exceeding 120 min¯^1^)	Compresses with a frequency of 100–120¯^1^	Tolerance-rate: 95–125¯^1^
Trained rescuers should perform mouth-to-mouth ventilation in the ratio of 30 compressions to 2 breaths	2 rescue breaths 30 chest compressions	ratio of chest-compression/breaths 30: 2	
	Open the airway Blow steadily to rise the chest	Gives effective breaths to rise the chest	
	Send someone for help and to find and bring an AED; switch on the AED	Sends to go for AED and switches on AED	
Bare chest	Attach the electrode pads on the victim’s bare chest	Pads are attached correctly	
Don’t stop until help arrives	Minimise interruptions in chest compression	Ensures continuous effective chest-compressions	No interruption of more than 10 s

#### Second step

We developed a summative assessment-checklist to score the outcome of the educational event and to compare the groups. Targeting a binary outcome for every examinee (pass/fail), the trial leader reduced the items stepwise generating pass-relevant items. Quality was ensured with respect to the two most common flaws in examinations, “construct underrepresentation” and “construct irrelevant variance” [10]. Construct underrepresentation was avoided by including only outcome relevant items and excluding items oversampling of one content area by double-scoring. According to current literature, we focused on chest-compression as the key determinate to improve survival and excluded mouth-to-mouth ventilation.

Construct irrelevant variance was achieved by excluding items with unreliable scoring or representing not mainly task-specific knowledge and skills of the examinee. The final checklist contained 15 Items of the formative assessment and eight of those, (Table 2) were relevant to pass the exam. The summative assessment was judged “passed”, when all eight items of the checklist were rated with “yes” by both examiners. If one item was judged “no” the overall assessment was judged as “failed”.

**Table 2:** Steps to develop summative assessment

Formative assessment	Steps to develop summative assessment				Summative assessment	
	Steps to reduce construct underrepresentation		Steps to reduce construct irrelevant variance			
Initial items	Outcome relevant?	Item part of other item scoring	Likley to be assessed reliably?	Item only testing knowledge and skills?	Relevant items	Specified dimension of performance
Speaks to the victim and shakes his shoulders	Yes	No	Yes	Yes	speaks to the victim and shakes his shoulders	
Calls for help	Yes	No	Yes	No		
Checks if the victim is breathing normally	Yes	No	Yes	Yes	checks if the victim is breathing normally	opens airway and comes close
Calls or sends to call 112	Yes	No	Yes	Yes	Calls or sends to call 112	
Starts chest-compression as soon as possible	Yes	No	Yes	Yes	Starts chest-compression as soon as possible	Within the first 30 s
Has the right compression point	Yes	No	Yes	Yes, with limitations	Has the right compression point	Center of the chest
Has the right position	Yes	Yes (compr.depth)				
Has the right compression depth	Yes	No	Yes	Yes	Has the right compression depth	5–6 cm depth, Mini-Anne clicking min. 4 of 5 compr.
Releases all the pressure from the chest	Yes	Yes (compr.depth)				
Compresses with a frequency of 100–120_^1^	Yes	No	Yes	Yes	Compresses with a frequency of 100–120_^1^	Tolerance-rate: 95–125_^1^
Ratio of chest-compression/breaths 30: 2	Questionable					
Gives effective breaths to rise the chest	Questionable					
Sends to go for AED and switches on AED	Yes	No	Yes	No		
Pads are attached correctly	Yes	No	Yes	No		
Ensures continuous effective chest-compr	Yes	No	Yes	Yes	Ensures continuous effective chest-compr.	No interruption of more than 10 s

### Statistical analysis

We used the intraclass correlation coefficient (ICC) to assess inter-rater reliability of the assessment between the different groups of assessors. Statistical analyses were performed with SPSS Version 21 (IBM SPSS Inc., Chicago, IL, USA) using a two-way-random-model (confidence-interval 95 %). ICC is rated moderate for values greater 0.5, strong for values greater 0.7, optimal for values greater 0.8 and excellent for values greater 0.9. To test independency of failure-rates from the assessor, we performed McNemar’s test with GraphPad Prism 5 (GraphPad Software, Inc., La Jolla, CA, USA) to compare failure-rates of every item between the different assessor-groups assessing the same school children. With respect to multiplicity, we used the Bonferroni-Method and the two-sided significance level was set at 0.6 % (0.05/number of tests: 0.05/8 = 0.0063) for every hypothesis to keep overall type I error at 5 %. Power-calculation was performed for the associated study and determined the required sample size of 371 school children per group (peer-led vs. professional-led group) [5]. We expected the sample size of main study as adequate to evaluate intraclass correlation coefficient (ICC) between professionals, medical students and peers with narrow confidence limits.

## Results

**Fig. 1:**
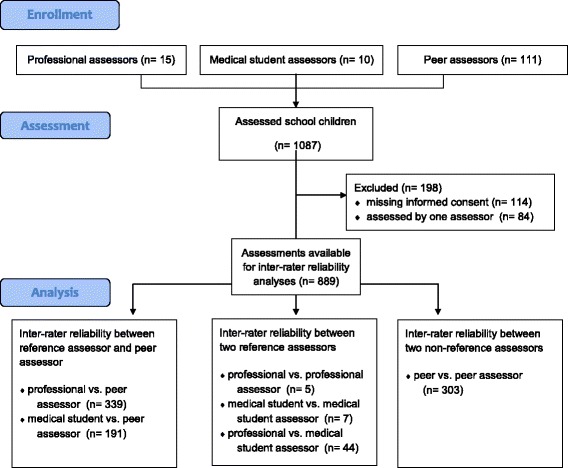
Flow-Chart: 111 peer-instructors, 10 medical students and 15 professional trainers evaluated 1087 school children with this checklist. 114 school children forgot their written informed consent and 84 were evaluated by only one assessor. Overall data of 889 summative-assessments were used to calculate inter-rater reliability

One hundred eleven peer-instructors, 10 medical students and 15 professional trainers evaluated 1087 school children with this checklist. Demographic data of the assessors are presented in Table 3. One hundred fourteen school children forgot their written informed consent and 84 were evaluated by only one assessor. Overall data of 889 summative-assessments were used to calculate inter-rater reliability (Fig. 1). Mean age of the assessed school children was 14 years, 51 % were female and 49 % male school children.

**Table 3:** Demographic data of the assessors

Characteristic	Professional-assessors	Student-assessors	Peer-assessors
Age – yr (S.D.)	34.5 (± 4.5)	25.2 (± 1.0)	16.2 (± 1.0)
Gender (male) – no. (%)	11/15 (73.3 %)	4/10 (40.0 %)	47/111 (42.3 %)
School – yr (S.D.)	12.8 (± 0.4)	13.2 (± 0.4)	10.7 (±0.8)
*Stadtteilschule* – no. (%)	2/15 (13.3 %)	0/10 (0 %)	55/111 (45.0 %)
College – yr (S.D.)	6.1 (±0.4)	5.1 (± 0.3)	–
Professional experience – yr (S.D.)	7.4 (± 4.1)	–	–

*SD* standard deviation

### Inter-rater reliability

Overall inter-rater reliability (Table 4) according to average measure of intraclass correlation coefficient (ICC) was 0.84 (*n* = 889, 95 % CI: 0.82 to 0.86). The number of comparisons between peer-peer assessors (*n* = 303), peer-professional assessors (*n* = 339), and peer-student assessors (*n* = 191) was high and demonstrated strong inter-rater reliability between peer- and professional assessors (ICC: 0.76; 95 % CI: 0.71 to 0.81), optimal inter-rater reliability between peer- and student-assessors (ICC: 0.88; 95 % CI: 0.84 to 0.91) and excellent inter-rater reliability between peer- and other peer-assessors (ICC: 0.91; CI: 0.89 to 0.93). The comparison student versus professional demonstrated moderate inter-rater reliability (ICC: 0.66; 95 % CI: 0.37 to 0.81) with a sample size of 44. The sample size for the comparison professional versus professional and student versus student was very low following inter-rater reliability (professional vs. professional: *n* = 5; ICC = 0.75; 95 % CI: −1.40 to 0.97/student vs. student: *n* = 7; ICC = 0.77; 95 % CI: −0.34 to 0.96). Agreement between peer- and student assessors was high. Disagreement (assessment judged as “failed” by only one of the two assessors) occurred comparably often in the group of peer- and student-assessors. The scoring of peer- and professional-assessors differed in 65 of 339 ratings. Peers rated the examinations of the school children more often as “failed” (*n* = 43) compared to professionals (*n* = 22). Professionals, compared to medical students, rated the examination of school children more often as “failed” (number of examinations only failed according to the scoring of professional-assessors: *n* = 7/student-assessors: *n* = 3).

**Table 4:** Inter-rater reliability

Assessors	Assessed school children (*n*=)	School children rated “passed” by both assessors (*n*=)	Intraclass correlation coefficient (ICC)	95 %-confidence-interval of ICC	School children rated “failed” only by peer-assessors (*n*=)	School children rated “failed” only by student-assessors (*n*=)	School children rated “failed” only by professional-assessors (*n*=)
All	889	309/889	0.84	0.82 to 0.86			
Peer/peer	303	124/303	0.91	0.89 to 0.93			
Student/student	7	1/7	0.77	−0.34 to 0.96			
Professional/professional	5	3/5	0.75	−1.40 to 0.97			
Peer/student	191	56/191	0.88	0.84 to 0.91	9	9	
Peer/professional	339	116/339	0.76	0.71 to 0.81	43		22
Student/professional	44	9/44	0.66	0.37 to 0.81		3	7

Peers and professionals rated the items “check responsiveness”, “correct compression point” and “no-flow-time” significantly different (*p*-value of McNemar’s test for “check responsiveness” = 0.004, “correct compression point” = 0.001 and “no-flow-time” < 0.001). The school children failed those items more often, when rated by peers than by professionals. Failure-rates of the other items did not differ between peer- and professional-assessors (Table 5). Failure-rates of the school children rated by medical students and professionals and those rated by students and peers differed not significantly according to McNemar’s test.

**Table 5:** Failure-rates for every item of the school children assessed by peer- and professional-assessors

Item	Failure-rate peer-assessorsn (*n* = 339)		Failure-rate professional-assessors (*n* = 339)		*P*-value of McNemar’s test
	no.	(%)	no.	(%)	
check responsiveness	31	9.1 %	22	6.5 %	0.004
check breathing	45	13.3 %	44	13.0 %	1.00
call 112	21	6.2 %	17	5.0 %	0.13
compress (start immediatly)	31	9.1 %	29	8.6 %	0.50
compress (correct point)	19	5.6 %	8	2.4 %	0.001
compress (correct depth)	57	16.8 %	53	15.6 %	0.13
compress (correct frequency)	88	26.0 %	94	27.7 %	0.03
compress (no -flow-time)	54	15.9 %	29	8.6 %	< 0.001

## Discussion

In the current study, we found a good inter-rater reliability between professional- and peer-assessors (ICC > 0.7) and optimal (ICC > 0.8) to excellent (ICC > 0.9) inter-rater reliability between peer- and other peer- or student-assessors, according to intra-class correlation coefficient (ICC) and the 95 %-confidence interval of ICC.

School children were more likely to pass the exam when rated by a professional than by a peer because they failed the items “check responsiveness”, “correct compression point” and “no-flow-time” more often, when rated by peers than by professionals. These results are in line with the current literature indicating examinees are more likely to pass the exam, when rated by professional-instructors than by (student-) peer-instructors [11, 12].

Though we can’t explain the differences on item level based on current literature, we assume that the peers rated the items “check responsiveness”, “correct compression point” and “no-flow-time more “text-book” based on definitions and metrics. Whereas professionals may have used their medical experience to judge items “yes” that aren’t necessarily “correct” based on guideline definitions but work out in a real-life.

Failure-rates of the school children rated by medical students and professionals did not differ significantly for the whole assessment. There was no systematic scoring difference between these two groups of assessors on single item level. Neither could we demonstrate a significant, systematic scoring-difference between student- and peer-assessors.

This study adds two important aspects to the current literature:

1. This study developed a context-appropriate assessment of hand-on BLS-skills of school children. It is reliable using professional instructors, medical students and peers as assessors. Additionally, the new assessment meets other performance-indicators like feasibility, cost effectiveness, specificity and fidelity [6]. Using low-cost assessment instruments (MiniAnne and smart-phones of the assessors) and including medical students as well as trained school children as assessors, we managed to assess up to 96 school children per hour (8 assessment-sites) at the end of one CPR-training-event at the schools.
2. The analysis of inter-rater reliability between the three assessor-groups (professionals, medical students and peers) demonstrated peers and medical students are capable of reliably assessing BLS skills. We could not show any systematic rating-difference between professional- and student-assessors. But failure-rates of school children rated by peers were higher. The severe examination by peers led to a low-risk of school children passing the exam unjustified when rated by peers.

There are several limitations of the study. First of all the dropout rate was high. One hundred ninety-eight assessments could not be included in the analysis because school children forgot their written informed consent or were only evaluated by one assessor. We cannot estimate the consequence of the missing assessments on the results. The development of the assessment-checklist and the assessor-training was performed by one professional-trainer only. Probably a consensus-process like the DELPHI-process [13] would have enforced the content-validity of the assessment checklist. The fact that we used frame-of-reference training for all assessors, the scoring of the assessment checklist is closely related to our reference-rater. This has to be considered before generalising the results.

The CPR performance assessed with MiniAnne was not compared to performance assessed with another instrument of assessment e.g. high-quality-feedback mannequin, thus allowing to estimate of dependency of pass-rates and reliability of assessors from the assessment instrument. The “clicking” sound of the MiniAnne did not only offer feedback for the assessors but also for the examinees and probably improved the observed compression quality. The influence of supportive behaviour of the assistant rescuer on performance of the examinee during the two-rescuer scenario could be reduced by using a “standardized” role-play for the assistant rescuer.

The summative assessment scores performance of compression-only CPR. According to current literature, we focused on chest-compression as the key determinate to improve survival. We excluded mouth-to-mouth ventilation from the summative assessment, because in the city of Hamburg the emergency medical service arriving time is in 79 % within 8 min and cardiac origin of arrest is very high [14]. In different locations mouth-to-mouth ventilation may be more important for patient outcome [15].

## Conclusion

Using this assessment and integrating peers (trained school children) and medical students as assessors gives the opportunity to assess hands-on skills of school children, with high reliability.
